# Antigen test swabs are comparable to nasopharyngeal swabs for sequencing of SARS-CoV-2

**DOI:** 10.1038/s41598-023-37893-5

**Published:** 2023-07-12

**Authors:** Sayf Al-Deen Hassouneh, Alexa Trujillo, Sobur Ali, Eleonora Cella, Catherine Johnston, Katherine C. DeRuff, Pardis C. Sabeti, Taj Azarian

**Affiliations:** 1 Burnett School of Biomedical Sciences, University of Central Florida, Orlando, FL USA; 2 Broad Institute of MIT and Harvard, Cambridge, MA 02142 USA; 3 Genomics and Bioinformatics Cluster, University of Central Florida, Orlando, FL 32816-2993 USA

**Keywords:** Virology, Pathogens

## Abstract

Viral genomic surveillance has been integral in the global response to the SARS-CoV-2 pandemic. Surveillance efforts rely on the availability of representative clinical specimens from ongoing testing activities. However, testing practices have recently shifted due to the widespread availability and use of rapid antigen tests, which could lead to gaps in future monitoring efforts. As such, genomic surveillance strategies must adapt to include laboratory workflows that are robust to sample type. To that end, we compare the results of RT-qPCR and viral genome sequencing using samples from positive BinaxNOW COVID-19 Antigen Card swabs (N = 555) to those obtained from nasopharyngeal (NP) swabs used for nucleic acid amplification testing (N = 135). We show that swabs obtained from antigen cards are comparable in performance to samples from NP swabs, providing a viable alternative and allowing for the potential expansion of viral genomic surveillance to outpatient clinic as well as other settings where rapid antigen tests are often used.

## Introduction

Coronavirus disease 2019 (COVID-19) has highlighted the critical public health role of continual testing and viral genomic surveillance for tracking emerging variants, understanding transmission, linking viral evolution to changes in disease epidemiology, designing and evaluating diagnostic tools, and forecasting vaccine efficacy in the context of viral diversity^1,2^. COVID-19 caused by severe acute respiratory syndrome coronavirus 2 (SARS-CoV-2) has led to approximately 760 million confirmed cases and 6.9 million deaths reported worldwide by the World Health Organization (WHO)^3^. SARS-CoV-2 genome evolution throughout the pandemic has led to the continual emergence of new variants with increased transmissibility, disease severity, and capacity for immune escape^4–6^. Since the first SARS-CoV-2 genome sequences were published in January 2020^7^, over 15 million sequences have been shared via the Global Initiative on Sharing All Influenza Data (GISAID) database^8^, and over 7 million nucleotide sequences have been deposited in National Center for Biotechnology Information (NCBI) (https://www.ncbi.nlm.nih.gov/sars-cov-2/) through 29 May 2023. The unprecedented effort to monitor SARS-CoV-2 viral evolution has permanently changed the approach to pathogen genomic surveillance worldwide.

SARS-CoV-2 genome sequencing approaches have most widely been applied to positive diagnostic samples from nucleic acid amplification testing (NAATs). The gold standard and most widely used NAAT is Real-Time Reverse Transcription Polymerase Chain Reaction (RT-PCR). As both viral sequencing approaches and RT-PCR directly amplify viral genomic material, the collection methods and reagents and downstream protocols overlap, making it a useful approach for genomic surveillance. This has been an effective approach since RT-PCR was the most widely used approach early in the pandemic.

The testing landscape, however, has shifted considerably over the course of the pandemic towards rapid diagnostic tests (RDTs), most commonly antigen-based lateral flow tests (LFTs). There are now more than 400 SARS-CoV-2 commercially available RDTs worldwide^9,10^, and several antigen-based LFTs are authorized for over the counter home testing through emergency use authorization (EUA) in the US^11^. Antigen-based assays detect specific viral proteins or the virus directly without PCR amplification steps^12^. The versatility of LFTs for broad application in schools, clinics, and home settings has significantly increased their use. Further, in an effort to increase COVID-19 detection, the US has made LFTs freely available through mail order, subsequently distributing over 270 million test kits as of March 2022^13^. The sensitivity of antigen-based LFTs is comparatively lower than NAATs, especially in cases of low viral load or asymptomatic infection^9,14–18^; however, when used within 5–7 days of onset among symptomatic individuals, the test can achieve 99.2% sensitivity and 100.0% specificity^19^. When compared to NAATs, LFTs perform well with viral loads corresponding to a RT-qPCR Ct value ≤ 33 cycles^20–22^.

As robust viral genomic surveillance hinges on acquiring positive cases through testing, changes to testing practice will impact surveillance efforts if laboratory workflows are not robust to sample type. The ability to use previously collected swabs from positive LFTs for genomic analysis would be of particular benefit. As testing practices in US and abroad continue to shift, a greater proportion of testing will be conducted via LFTs. The ability to sequence from LFTs will allow researchers to obtain representative viral samples spanning the geographic and epidemiological scope of the pandemic, as viral genomic surveillance efforts continue throughout the subsequent phases of the response. Further, more LFT testing is likely to occur outside of the healthcare setting. This change could significantly reduce available samples for viral genomic surveillance and skew the available samples to only those tests performed in a clinical setting, which would result in a bias toward more severe cases. Capturing samples from LFTs would expand the representation of genomic surveillance. To this end, we compared the ability to use swabs collected from LFTs for viral genome sequencing to nasopharyngeal (NP) swabs used for NAATs. Primarily, we sought to determine whether the extraction reagent or other component of sample processing used for BinaxNOW COVID-19 Ag Card testing disrupted the ability to perform SARS-CoV-2 genome sequencing.

## Results

**Figure 1:**
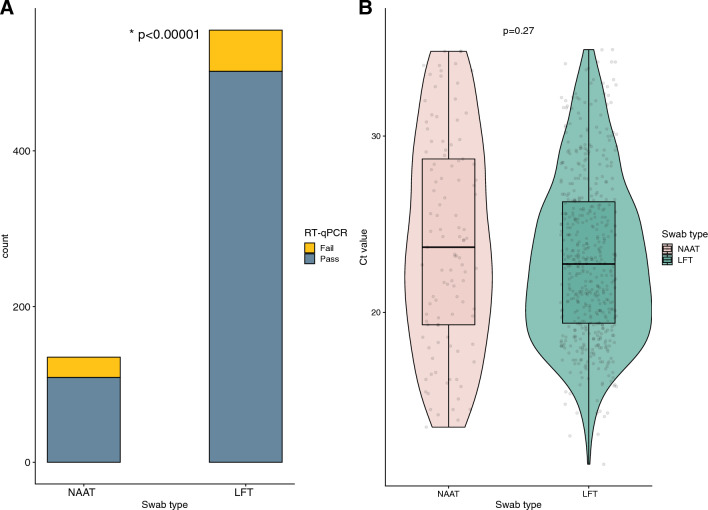
Plots displaying the comparison of sequencing quality metrics between NAAT and LFT swabs. (**A**) Bar plot of the swab type count by RT-qPCR outcome. (**B**) Samples with detectable virus according to the swab type by Ct value. Corresponding p-values are reported in each panel.

Of the 690 samples, 611 had detectible virus in the sample based on RT-qPCR Ct values after RNA extraction. There was a significant difference between NAAT samples and LFT that failed to amplify with a greater proportion of LFT samples successfully extracted (80.7% N = 109 NAAT samples vs 90.5% N = 502 LFT, p < 0.00001) (Fig. 1A). Among the samples that had detectable virus, we compared the RT-qPCR Ct values and found no significant difference between NAAT and LFT samples (median of 21.7 for NAAT and 21.9 for LFT, p = 0.27) (Fig. 1B).

**Figure 2:**
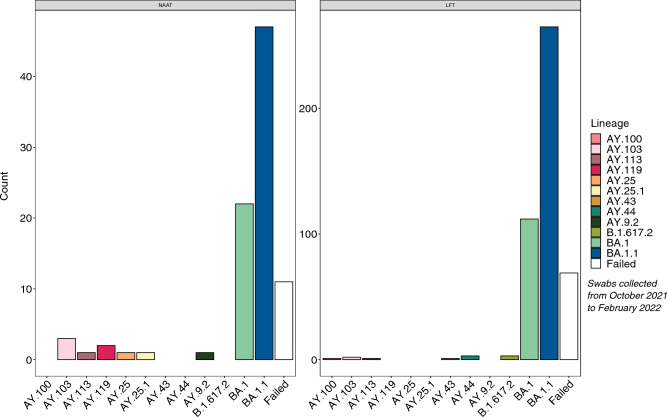
Lineage distribution for the samples sequenced stratified by swab type. Plot shows the distribution of the various SARS-CoV-2 variant lineages for each swab-type.

Using a cut-off Ct value of ≤ 30, 519 samples (78 NAAT and 390 LFT) were moved forward to viral genome sequencing. Subsequently, we found that there was no significant difference between NAAT vs LFT samples in the proportion that failed sequencing (8.1% NAAT vs 10.3% LFT, p = 0.48) and only a moderate significant difference in median sequencing coverage (median of 183 for NAAT vs 199 LFT, p = 0.0018) (Supplementary Fig. 1A). The lineage assignments for sequenced samples are shown in Fig. 2. Most samples (96.2%) were assigned to the SARS-CoV-2 Omicron variant (BA.1 and BA.1.1).

Comparing the time from sample collection to RNA extraction identified that the time to extraction was significantly shorter for LFT samples due to the logistics of our sample acquisition process (median of 20 days for NAAT vs 6 days for LFT, p < 0.00001) (Supplementary Fig. 1B). To assess the impact on time to extraction on outcomes, we compared results for each sample type independently. We did not find any significant difference in time to extraction for passing or failing outcomes for NAAT swabs (median of 19 days for pass vs 23 for failed, p = 0.15) (Supplementary Fig. 1C) or LFT swabs (median of 6 days for pass vs 7 for failed, p = 0.16) (Supplementary Fig. 1D).

Despite our lack of association of time to extraction on sequencing outcomes, we still aimed to rule out the effect of difference in time to extraction between NAAT and LFT swabs; we thus performed a sub-analysis by down-sampling based on time to extraction. We limited the subsample to samples with a time to extraction from 14 to 21 days. This resulted in 41 NAAT swabs and 44 LFT swabs, of which 35 NAAT and 38 LFT had detectable Ct values with no significant difference in failure by swab type (p = 0.6). After subsetting the data, the median time to extraction for both NAAT and LFT swabs was 16 days with no significant difference (p = 0.352) (Supplementary Fig. 2A). Further, there was no significant difference in time to extraction for samples that did not amplify for NAAT or LFT swabs (Supplementary Fig. 2B). For the subsample, RT-qPCR Ct value were again not significantly different between NAAT and LFT swabs (median of NAAT 19.9 vs 20.9 for LFT, p = 0.404) (Supplementary Fig. 2C). Using a cutoff Ct value of ≤ 30, 35 (85%) NAAT and 38 (86%) LFT samples moved forward to sequencing. There was also no significant difference in the proportion of samples from the subset that failed sequencing or difference in sequencing coverage among those successfully sequenced (Supplementary Fig. 2D). The statistical significance of the sub-analysis was unchanged when bootstrapping the sub-groups to generate groups of 100 for each comparison leading us to believe that the smaller sample sizes were not obfuscating significant changes.

To further examine the effect of time to extraction on coverage and RT-qPCR values, we performed a Spearman’s rank-order correlation analysis. We did not identify any significant correlation between time to extraction and RT-qPCR CT values in the total data (R = − 0.02, p = 0.65) or the subset data (R = 0.09, p = 0.52). There was also no significant correlation between time to extraction and coverage in the total dataset (R = − 0.02, p = 0.7) or the subset data (R = − 0.22, p = 0.1).

## Discussion

By sequencing samples derived from NAAT NP swabs and samples derived from positive BinaxNOW COVID-19 Ag Card swabs and comparing the proportions of successful sequencing, genome coverage, and RT-qPCR Ct values, we demonstrate that extraction reagents and sample processing do not significantly impact the ability to recover SARS-CoV-2 viral genomes. This suggests that positive LFT swabs could serve as a viable alternative for genomic surveillance. When comparing the sequencing results from LFT cards to NAAT swabs, there was no significant difference between the proportion of failed samples, genome coverage, or RT-qPCR Ct values. We did observe that NAAT samples had a significant, but marginal, longer time to extraction than the LFT samples. However, we did not observe a significant correlation between time to extraction and genome coverage or RT-qPCR Ct values, which implies that difference in time to extraction does not have an overt impact. These findings indicate that sequencing of positive LFT swabs could compliment traditional sequencing of NAAT swabs while yielding very similar sequencing quality. The increased ability to extract and amplify viral RNA from LFTs is consistent with their test performance, as previous studies have shown that they are more likely to report positive results with higher viral loads corresponding to RT-qPCR Ct values < 30. This may partially explain the high success in viral genome sequencing from positive LFT swabs.

The ability to use swabs from RDTs for SARS-CoV-2 genome sequencing is important for future surveillance efforts. As RDTs such as the BinaxNOW COVID-19 Ag Cards become more accessible and ubiquitous, NP swabs will become increasingly limited to a clinical setting where NAATs are routinely employed. This change could potentially bias the genomic surveillance data towards more severe cases that require clinical intervention. Capturing samples from RDTs performed in the home or clinic setting would enable us to generate surveillance data that is more representative. In addition, by using clinical excess from previously collected swabs, we also eliminate the need for collection of a second swab, which can simplify IRB protocols for clinical studies and increase study participation. The future of over-the-counter RDTs will depend on the course of the pandemic. While the COVID-19 public health emergency declaration recently expired in May 2023, the EUA allowing their use remains in place. Henceforth, manufacturers will need apply for formal FDA approval for continued use. However, it seems that the widespread use of RDTs during the pandemic could signal a paradigm shift for the availability of at-home and clinic-based infectious disease testing, and we feel that our findings may inform surveillance strategies for epidemiologically similar pathogens for which RDTs are currently employed. Undeniably, the availability of over-the-counter RDTs has provided agency to the public, allowing individuals to use testing to manage their personal risk and aid in decision making. Home testing could conceivably be expanded to other respiratory pathogens such as influenza virus or respiratory syncytial virus (RSV), which are commonly diagnosed in the outpatient setting using rapid antigen tests. With this possibility in mind, we must rethink the future of viral genomic surveillance so that sampling of cases in the community remains robust.

One possible solution for obtaining samples from at-home testing would be to partner with the government’s free at-home COVID-19 test program to provide a subsample of recipients with prepaid postage and mailing containers with viral transport media (VTM) that could be used to send positive samples to sequencing centers. Remuneration could also be considered to incentivize participation. While at-home storage and transport conditions may vary considerably, direct-to-consumer genetic testing through companies like 23andMe and AncestryDNA provide a model for collection and transport of samples with the intended application of sequencing. Further, several studies have extensively evaluated the stability of SARS-CoV-2 RNA in a number of storage and transport conditions with and without transport media and cold storage. In the study by Alfaro-Nunez et al., they found that viral RNA remained stable on dry non-buffered swabs for up to 26 days when left at room temperature^23^. Similar studies found that qRT-PCR Ct values remained stable among samples stored in phosphate-buffered saline or VTM at room temperature for up to 28 days regardless of viral loads^24–27^. While these studies have largely focused on qRT-PCR performance, the robustness of SARS-CoV-2 genome sequencing has been demonstrated through the ability to successfully reconstruct viral genomes from seemingly complex or low-quality samples including wastewater and environmental surfaces^28,29^. With the current level of at-home testing, even a modest sequencing failure rate of samples collected through a mail-in program would significantly improve community-based genomic surveillance. In the outpatient healthcare setting, primary care clinics performing RDTs are equipped to collect and store samples as described in this study and would provide a viable source for community-based sampling much like CDC’s U.S. Outpatient Influenza-like Illness Surveillance Network (ILINet).

Our study is not without limitation. Due to the observational nature of our study, we are not able to directly compare sequencing success of different sample types from the same participant. Furthermore, we did not consider vaccine history or disease severity which may vary between settings (i.e., NAAT: hospital, RDT: clinic); however, the study population from which these samples were obtained was generally healthy and likely experienced mild disease. As a note, samples that fail sequencing may be due to technical errors in library preparation; however, we expect this effect to be independent of swab type. Further, we report a significant difference in the time to RNA extraction between the two groups. To mitigate this issue, we conducted our analysis using a sub-set of the total data in which the time to extraction was between 14 and 21 days. This subset resulted in a similar number of samples between the two groups with similar time to extraction. Finally, in our study, positive BinaxNOW COVID-19 Ag Card swabs were removed from the card, placed in transport media, and stored in a clinic refrigerator until transport to the laboratory. While this protocol may function in clinic and outpatient settings, it may not be well suited for at-home testing, leaving the question of real-world viability unanswered. Indeed, we did not systematically evaluate the effect of variation in transport and storage conditions on viral genome sequencings; however, the previous studies described above have demonstrated that viral RNA stability is robust to storage duration and condition^23,27,30^. While subsequent studies using parallel sampling from the same individual or a variety of currently marketed RDTs could resolve these limitations, we believe our current study demonstrates the ability to successfully sequence SARS-CoV-2 from swabs used for LFTs. Overall, we show that sequencing LFT swabs is not only possible, but also results in comparable RT-qPCR Ct values, genome coverage, and sequencing failure rates. These findings provide the foundation for community-based viral genomic surveillance, which will allow public health to maintain representative sampling cases as we continue pandemic mitigation efforts.

## Materials and methods

### Collection of samples|participant recruitment

A total of 690 testing swabs were collected from NP samples from NAAT positive tests performed on the BioFire Torch using the Respiratory 2.1 panel (hereon referred to as NAAT, N = 135) or from positive BinaxNOW rapid antigen LFTs (N = 555). NP swabs were collected from children seeking care at a local hospital in Orlando, FL between October 2021 and February 2022. Positive NP NAAT swabs were placed in Zymo Research DNA/RNA shield VTM and stored at 4 °C at the healthcare facility until weekly scheduled pickup. The samples were then transported to the research laboratory in a Styrofoam cooler and ice following the U.S. Department of Transportation Hazardous Materials regulations and subsequently stored at 4 °C until RNA extraction. LFT swabs were mainly collected from college-aged individuals seeking care at university student health service clinic during the same period, from October 2021 to February 2022. BinaxNOW rapid antigen LFTs were preformed according to the manufacture, which requires the anterior nares swab to be inserted directly into the test card. After identification of positive specimens, swabs were removed from the test cards and placed in Zymo Research DNA/RNA shield and stored at 4 °C in the clinic until daily pickup. Swabs were transported by courier to the research laboratory, which was located adjacent to the clinic, and stored at 4 °C until RNA extraction.

### SARS-CoV-2 RNA extraction and RT-qPCR

RNA extraction for all samples was preformed using the QIAamp 96 virus QIAcube HT kit automated platform. Our RT-qPCR reactions were carried out in a 10 μL reaction using 4 × TaqPath master mix (Thermo Fisher Scientific, Massachusetts, USA), 0.25 μM each of 2019-nCoV_N1(CDC) qPCR probe (5′-FAM-ACCCCGCATTACGTTTGGTGGACC-BHQ1-3′), forward primer (5′-GACCCCAAAATCAGCGAAAT-3′), and reverse primer (5′-TCTGGTTACTGCCAGTTGAATCTG-3′), 4.25μL of molecular-grade H_2_O, and 2.5μL of template RNA. RT-qPCR was performed on a CFX Opus 96 instrument (Bio-Rad Laboratories, Hercules, California, USA) with the following conditions: UNG incubation at 25 °C for 2 min; reverse transcription step at 50 °C for 15 min, followed by polymerase activation at 95 °C for 2 min, and finally, 35 cycles of amplification at 95 °C for 15 s and 55 °C for 30 s. All samples were run in duplicate, including positive and no template controls.

### SARS-CoV-2 viral genome sequencing

Samples with RT-qPCR Ct values ≤ 30 were selected for sequencing. Samples were prepared and sequenced according to the Oxford Nanopore Technologies Midnight RT-PCR expansion pack (EXP-MRT001) along with the Rapid Barcoding Kit 96 (SQK-RBK110.96) protocol. In brief, viral cDNA was reverse-transcribed, followed by tiled PCR amplification, rapid barcode ligation, pooling, and SPRI bead clean-up. Libraries were sequenced using flow cells (R9.4.1) with the GridION. Base-calling and demultiplexing were performed in real-time using the GridION software. The assembly was performed in two steps (using default parameters) following the ARTIC Network bioinformatics protocol (https://artic.network/ncov-2019/ncov2019-bioinformatics-sop.html). The gupplyplex script was used for quality control and filtering of reads (fragments 1000–1500 bp) followed by assembly with the MinION pipeline, using medaka to call variants, with Wuhan-Hu-1 reference (GenBank accession number MN908947.3). We then used the *pangolin* software tool to assign the lineage of each sample.

### LFT vs NAAT performance comparison

To evaluate the suitability of samples obtained from positive rapid antigen tests for use in viral genome sequencing, we compared viral RNA extraction, RT-qPCR, and sequencing success to samples collected from the clinical excess of positive NAATs. We first assessed for statistically significant differences between the date of collection and date of viral RNA extraction between the two samples. We then compared the frequency of samples that failed to amplify during RT-qPCR and the resulting cycle threshold (Ct) values among those that amplified. The Ct value is inversely proportional to the amount of viral target in the sample—lower Ct values are associated with a greater quantity of virus and higher values are associated with a lower quantity. Last, we assessed sequencing success (failed samples, viral genome coverage, and genomes passing sequencing QC) between the two groups. Chi squared statistic was used to compare frequencies between categories (e.g., pass/fail for NAAT vs LFT) and the Mann–Whitney *U* test^31^ was used to determine if the values between two groups were significantly different sizes. Additionally, for the sub-analysis assessing the potential association of time to extraction on sequencing outcomes, we tested the robustness of the Mann–Whitney *U* test for our sample size by bootstrapping the sub-groups (41 and 44, respectively) to generate two groups of 100 for each comparison and re-running the statistical test and we found that the significance, as defined by a p-value < 0.05, was not impacted. All statistical analysis was performed using python 3.10.2^32^. All visualizations were produced using Rstudio running v 3.6.0^33^.

### Ethics statement

This study was reviewed by the University of Central Florida Institutional Review Board and received a non-human subject determination.

## Supplementary Information

Supplementary Figures.

Supplementary Table 1.

## Data Availability

All data produced in the present study are available upon reasonable request to the authors**.** The datasets generated and/or analyzed for this current study are available in GISAID and the accession ID’s are listed in Supplemental Table 1 along with additional metadata.
