# Supplementary material for: Antigen test swabs are comparable to nasopharyngeal swabs for sequencing of SARS-CoV-2
Source: Sci Rep. 2023 Jul 12;13:11255. doi: 10.1038/s41598-023-37893-5 (PMC10338537; doi:10.1038/s41598-023-37893-5)
Supplement: Supplementary file 1 — Supplementary Figures. [file 41598_2023_37893_MOESM1_ESM.pdf]

Supplementary Materials for
Antigen test swabs are comparable to nasopharyngeal swabs for sequencing of SARS-CoV-2

Sayf Al-Deen Hassouneh, Alexa Trujillo, Sobur Ali, Eleonora Cella, Catherine Johnston, Katherine C. DeRuff, Pardis C. Sabeti, Taj Azarian*

*Corresponding author. Email: taj.azarian@ucf.edu

This PDF file includes:

Figs. S1 and S2

Fig. S1.

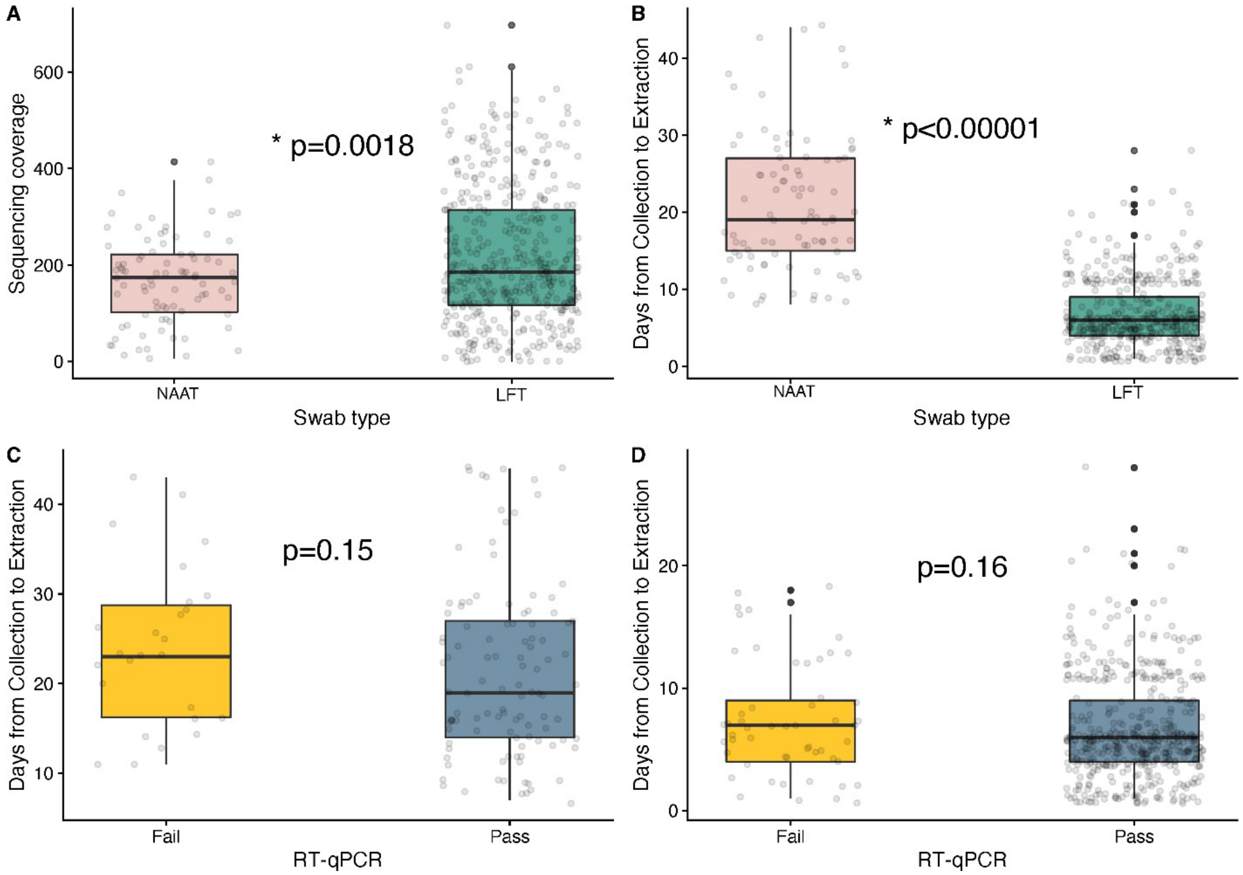

Samples with a Ct value <30. Samples according to the swab type by sequencing coverage (panel A) and by time of extraction (days) (panel B). Comparison of time of extraction according to RT-qPR outcome in NAAT (panel C) and LFT (panel D) swabs. Corresponding p-values are reported in each panel.

Fig. S2.

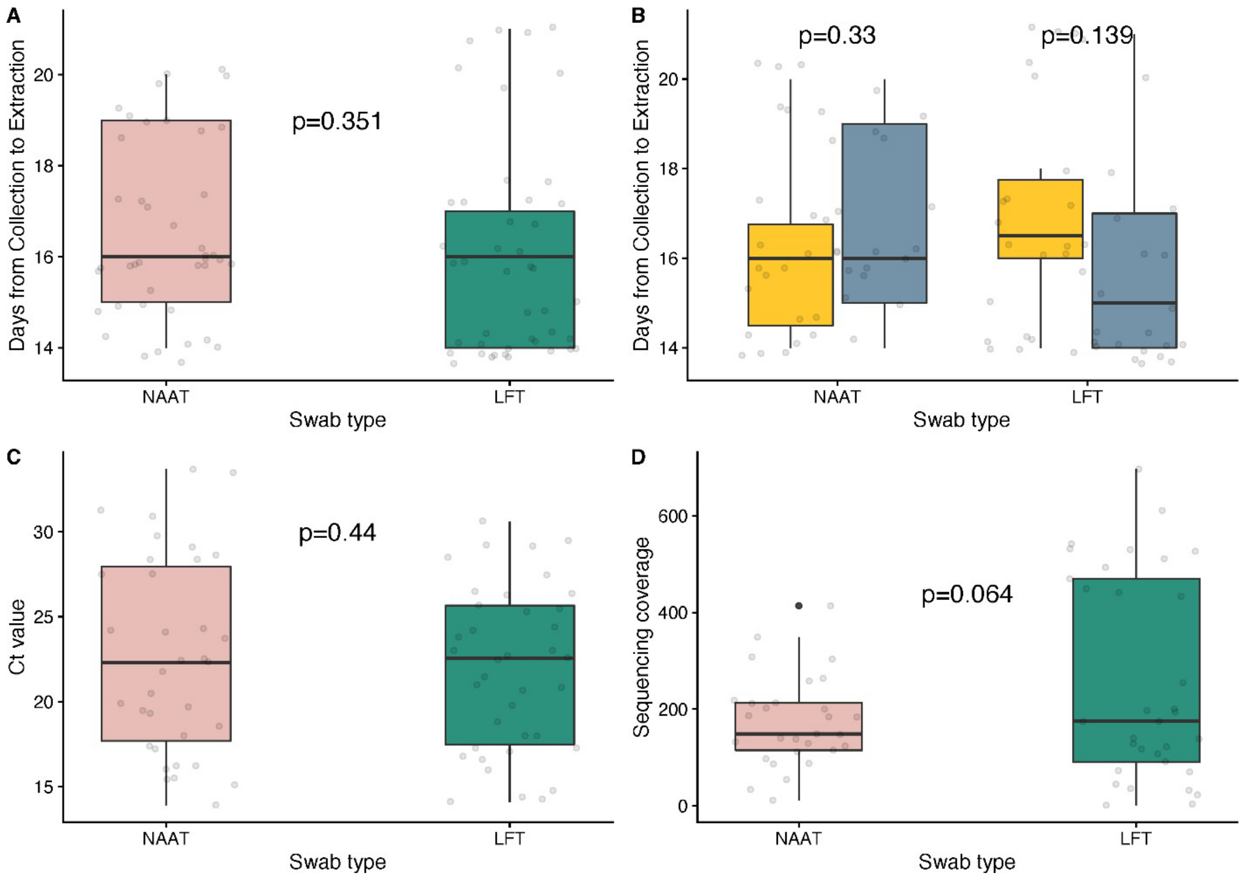

Sub-analysis of samples with a time of extraction from 14 to 21 days. Samples according to the swab type by days from collection to extraction (panel A). Comparison of time to extraction according to the swab type by RT-qPCR outcome (panel B). Samples according to the swab type by Ct value (panel C) and by sequence coverage (panel D). Corresponding p-values are reported in each panel.
